# The Democratic Ideal

**Published:** 1920-04-10

**Authors:** 


### THE COLLEGE ELECTIONS.

### The Democratic Ideal.

It is often pointed out, in connection with the College of Nursing, that its Constitution is democratic, and that it is within the power and discretion of its members to elect whomsoever they choose to act on the Executive Committee. While this is literally true, there is, I venture to maintain, something lacking. The Constitution is all right, but, somehow, the democratic ideal does not (in my opinion at all events) appear to be realised. All lovers of the College, Council, and members, should endeavour, first, to discover if there is such a defect as I suggest; and, secondly, if there is, to find a remedy.

The Democratic Ideal.

What is a democratic ideal?

There are many people even in the twentieth century, who are afraid of the word "democratic." They think that it is the beginning of an inclined plane which ends in anarchy and Bolshevism, and they do not hesitate to state that in a democratic community "Jack is as good as his master." This, of course, is quite untrue. All that is implied by the term democratic government is that the elector chooses his representative. And, although the facts may appear to be against me, where the College is concerned, nevertheless I cherish the conviction that things are not what they seem. The Nurse votes, not for a policy, but for an individual.

The Case Illustrated.

For the past four weeks, may I point out, by way of illustration, I have been advocating the claims of Trained Nurses to employment as Health Visitors. I have no means of ascertaining to what extent the College Executive endorse my arguments, or what efforts they are prepared to make in order to secure a suitable revision of the Regulations. Their attitude, whatever it may be, is their policy.

I quote this, as I say, by way of illustration. There are other matters. There is, for example, the eight-hour day. So far as I am aware, the Executive of the College have not committed themselves to any expression of opinion on this subject. And, here again, their attitude is their policy.

Now these are questions of great magnitude. I might call them, without exaggeration, burning questions. I might mention many more, but I am not now engaged in cataloguing grievances. My object is rather to cause clearness of thinking on the subject of voting at elections, according to a democratic ideal. I have a recollection of a press note of triumph, following a College election, somewhat to the effect that all the nominated candidates were elected. I must confess that I did not see any cause for elation. The achievement was regarded as demonstrating unison of thought. It seemed to me an election conspicuous for want of thought. In every instance a name was put up for election, and in no instance was the nurse presented with alternative policies.

Compare with this a parliamentary election. Before women won the suffrage, parliamentary candidates found it unavoidable to express in the clearest terms their attitude on this question. It did not matter what their names were, or what they had accomplished for the nation as statesmen. If they were anti-suffrage, all the forces that could be availed of were ranged against them, and the elected candidate, whoever he happened to be, knew that he represented the majority of his constituents. The choice that had been made, wise or foolish, was the people's choice.

I call this the democratic ideal. And I am convinced that the defect to which attention is now called militates against the growth and the strength of the College.

The nursing world is a large one, but compared with other communities it is small; far too small to harbour factions, without inflicting serious damage on its members. When the war broke out the kingdom of the nurse was in a parlous condition. It was a kind of Donnybrook Fair. There were loud and clamouring voices, bitter enmities, will-o'-the-wisp schemes—and a complete absence of any policy that made for welfare. The result was that the rank and file remained scandalously overworked, and scandalously underpaid, with no ultimate outlook but the almshouse.

Then came the College. It was a great effort—a war effort. I have always held, and I still hold, that in its conception the College is an ideal institution. Here are the principles which appealed and appeal to me.

(i.) The Trained Nurse was granted full power to choose her representative.

(ii.) The avowed aim of the College was to gather into one great sisterhood the Trained Nurses of the Empire, and to promote their social and economic welfare.

(iii.) The word "College" indicated a high educational outlook.

Noble ideals every one.

Now what do I find? I am afraid that there are incipient symptoms of apathy apparent. The slogan of progress is not being sounded, and factions are growing. I repeat, there is no room for faction in the Nurses' commonwealth. What is wanting is one great union with a forward policy. I am as convinced as I am of my own existence that the British public will turn down no reasonable demand. But such demand must be prepared and presented. The Nurse looks to the College Executive for its programme, a manifesto of things to be achieved. The names matter somewhat, but the policy and the programme matter more. They are, in truth, practically everything. And in these days the electorate in every civilised community regard it as a matter of course that they should be kept fully in touch with what their representatives do and intend to do on their behalf.

If this is done there will be little cause for fear that the Trained Nurse will be scrapped.

IERNE.

